# Short-term mercury exposure in tilapia (*Oreochromis niloticus*) at different salinities: impact on serum osmoregulation, hematological parameters, and Na^+^/K^+^-ATPase level

**DOI:** 10.1016/j.heliyon.2020.e04404

**Published:** 2020-07-11

**Authors:** Kiki Syaputri Handayani, Bambang Irawan, Agoes Soegianto

**Affiliations:** Department of Biology, Faculty of Science and Technology, Universitas Airlangga, Jl Mulyorejo, Surabaya, Indonesia

**Keywords:** Physiology, Toxicology, Environmental pollution, Water pollution, Environmental toxicology, Zoology, Animal physiology, Tilapia, Mercury, Salinity, Ecology, Osmoregulation

## Abstract

The objectives of this study were to analyze and compare the effects of mercury (Hg) exposure on osmoregulation and hematological responses in East Java strain tilapia (*Oreochromis niloticus*). Fish were exposed to 0, 0.1, and 1 mg L^−1^ Hg at 0, 5, 10, and 15 g L^−1^ salinities, and serum osmolality (SO), ion level, hematological parameters, and sodium (Na^+^)/potassium (K^+^)-ATPase (NKA) levels in the gills and kidney were assessed after 96 h of exposure. SO significantly increased in fish exposed to Hg at 15 g L^−1^ salinity compared with those exposed at 0, 5, 10, and 15 g L^−1^ salinities, but SO did not significantly increase in fish exposed to Hg at 5 and 10 g L^−1^ salinities compared with those exposed at 0 g L^−1^ salinity. At 15 g L^−1^ salinity, the Na^+^ level was significantly different from that at 0, 5, and 10 g L^−1^ salinities. The chloride ion level significantly increased only at 15 g L^−1^ salinity. Furthermore, the K^+^ level was significantly different at 10 and 15 g L^−1^ salinities from that at 0 and 5 g L^−1^ salinities. Hematocrit and hemoglobin levels and red blood cell and white blood cell (WBC) counts were not significantly different among all salinities. At 15 g L^−1^ salinity, the NKA level in the gills was significantly different from that at 0 g L^−1^ salinity, but in the kidney, there was no difference among all salinities. These data provide useful information for future reference and aquaculture practices to reduce Hg effects on tilapia. In conclusion, higher salinity reduced the effect of Hg on the K^+^ level and WBC count in tilapia.

## 1 Introduction

Marine pollution by metals has increased in recent years due to the increase in the global population and industrial development. Estuarine zones are affected more by this problem than the oceans because of their proximity to the sources of pollution [1]. Water harbors all types of biotic and abiotic components, including metals essential for life processes. Many of these metals such as copper (Cu), iron (Fe), and zinc (Zn) are essential micronutrients but can become toxic at concentrations higher than that required for normal growth [2]. Other metals such as cadmium, mercury (Hg), and lead have no known roles in living organisms and are toxic even at very low concentrations [3]. The Minamata disaster that occurred in Japan in the 1950s has been the worst Hg-related incident in history. This incident has raised enormous concerns about Hg contamination of aquatic animals [4].

Metals concentrations in fish are important with respect to both environmental management and human consumption [5]. Metals accumulation in fish depends on many factors including their bioavailability and possible pollutant source [6], region and time of fishing, sex, size, species, nutrition, concentration in water, exposure time to metals, and other environmental factors (such as salinity, pH, and water temperature) [7]. Teleosts need to have highly efficient ionoregulatory/osmoregulatory mechanisms to maintain their body fluid homeostasis, which is necessary for normal physiological functioning. The mechanisms underlying NaCl secretion through the seawater (SW) teleost gill epithelium are better understood than those underlying NaCl absorption through the freshwater (FW) teleost gill epithelium. Euryhaline teleosts exhibit adaptive changes in sodium (Na^+^)/potassium (K^+^)-ATPase (NKA) activity following salinity changes [8, 9]. In gill epithelial cells, most NKA detected by immunostaining is localized in mitochondria-rich cells, i.e., chloride cells or ionocytes [10].

Evaluation of the chemical quality of water and aquatic organisms while studying metals effect is valuable for human life [11, 12]. In several studies, fish were selected as an experimental model to assess the effect of Hg on an organism, such as oxidative stress profiles and mercury accumulation [13, 14, 15, 16, 17, 18, 19]. Zhou and Wong [20] studied mercury concentrations in fish tissue correlated with the Hg levels in their ambient environment, and sediment seemed to be the major source of Hg accumulating in fish from the Pearl River Delta in Hong Kong. Soegianto et al. [21] measured serum osmolality (SO), ion levels, and hematological parameters in East Java strain tilapia (*Oreochromis niloticus*) and found that white blood cell (WBC) count was slightly increased at a higher salinity.

In this study, the East Java strain tilapia was selected as a model fish species not only because of the presence of Hg from gold mining activities in East Java but also because tilapia is one of the most widely consumed fish. They can encounter a wide range of salinity and other environmental conditions during their lifetime [22, 23, 24]. According to Spiegel et al. [25], there are also some sites of Hg sources in Indonesia caused by small-scale gold mining activities, e.g., in South Kalimantan, Central Kalimantan, Central, East and West Java, and North Sulawesi.

Hematological parameters are frequently used to evaluate the effect of metals on fish [26, 27]. In fish, the gill is the major organ responsible for osmoregulation and ionoregulation [10, 28]. Many studies have reported that different tilapia strains can grow well in water salinity ranging from 0 to 32 g L^−1^ [29, 30, 31]. As a consequence of the fluctuations in salinity, tilapia typically possess highly developed mechanisms for osmoregulation and ionoregulation to maintain osmotic and ionic homeostasis in their body [32]. Therefore, we experimentally explored the physiological and hematological effects of short-term Hg exposure on tilapia maintained at different salinities by measuring SO, serum ion levels, and hematological parameters after 96 h exposure. Gill NKA level was also analyzed after 96 h exposure. This study further enhances our comprehensive understanding of tilapia physiology when exposed to sublethal concentrations of Hg at different salinities.

## 2 Materials and methods

### 2.1 Fish acclimation

Nile tilapia (East Java strain, local name: Jatimbulan), with an approximate total length of 15.8 ± 0.7 cm and a body weight of 69.8 ± 1.1 g, were obtained from the Freshwater Aquaculture Development Unit of East Java (UPT-PBAT) in Pasuruan, East Java, Indonesia. After transporting the fish to the laboratory rearing facility of Airlangga University, the animals were maintained in 250 L fiber tanks filled with freshwater (FW) for 7 days under continuous aeration and a natural light system. Then, the fish were acclimated to different salinities (0, 5, 10, and 15 g L^−1^ with a 5 g L^−1^ daily increase in salinity to avoid osmotic shock) for 14 days under laboratory conditions before the start of the experiment. The fish were fed daily with commercial dried fish food pellets (30% protein, 3% fat, and 4% fiber) (Takari, Sidoarjo, Indonesia). Fecal and other waste materials were siphoned off daily to reduce the ammonia content in the water. Temperature was measured using a glass thermometer (°C), pH with a pH meter (Hanna Model HI 98150; Beijing, China), and dissolved oxygen (DO) with a DO meter (Lutron DO 5510, Taiwan). Temperature, pH, and DO values were 27.5°C–30 °C, 7.8–8.2, and 7–7.7 mg L^−1^, respectively.

### 2.2 Hg stock solution

A stock solution of Hg (1000 mg L^−1^) was prepared by dissolving 1.3539 g HgCl_2_ (Merck, Germany) in 1 L deionized water and it was stored in borosilicate glass containers. The pH of the stock solution was adjusted to approximately 7 to prevent metal adsorption on the wall of the container by adding HNO_3_ or NaOH (0.1 mol L^−1^) [33]. Based on the LC_50_ values obtained with the acute toxicity test (unpublished data), a control (without Hg in the test media), a lower sublethal concentration (0.1 mg L^−1^), and a higher sublethal concentration (1 mg L^−1^) of Hg were selected for the *in vivo* exposure experiment.

### 2.3 *In vivo* exposure experiment

After acclimation, 120 healthy fish were randomly selected from the holding tank and distributed among 24 tanks (n = 5 per tank). Each tank contained 40 L selected testing media: 1, 0.1, and 1 mg L^−1^ Hg and the control (without Hg) at 0, 5, 10, and 15 g L^−1^ salinities. There were two tanks per concentration. Exposure was continued for 96 h, and blood and tissue sampling was performed after 96 h exposure using 5 fish for each treatment. Wastewater containing the Hg was moved to a metals waste tank after the experiment. The experiments were conducted in accordance with the principles and procedures approved by the ethics committee of the Institutional Animal Care of Research Institute of Universitas Airlangga (314/UN3.14/LT/2019 signed by Prof. Hery Purnobasuki).

### 2.4 SO, ion levels, hematological parameters, and NKA level

Fish were anesthetized with 200 mg L^−1^ clove solution prior to blood sampling according to a method described previously [21]. Blood samples from each fish were obtained by puncturing the heart using a nonheparinized syringe. Then, blood samples were added to Vacutainer blood collection tubes containing 10.5 mg tripotassium ethylenediaminetetraacetic acid as an anticoagulating agent for the assessment of hematological parameters and to microtubes for the assessment of SO and serum ion (Na^+^, Cl^–^, and K^+^) levels. Blood samples from nonheparinized tubes were centrifuged at 5,000 rpm for 10 min at 4 °C to separate the blood serum and cells. Sera were used to assess the SO, Na^+^, Cl^–^, and K^+^ levels. SO was measured using an automated freezing point depression osmometer (Fiske® 210 Micro-Sample Osmometer, Norwood, Massachusetts USA) and expressed as mOsm kg^−1^.

Serum Na^+^, Cl^–^, and K^+^ levels were measured using an electrolyte analyzer (SpotChem EL SE-1520, Kyoto, Japan). Blood samples from the Vacutainer blood collection tubes were aspirated directly using an automated hematology analyzer (SFRI Blood Cell Counter 33, Jean d'Illac, France) to assess hematological parameters, i.e., RBC count, Ht level, and Hb level. SFRI Blood Cell Counter 33 uses electric resistance detection (impedance technology) with hydrodynamic focusing to measure the RBC count and Ht level. The Hb level was measured photocolorimetrically using sodium lauryl sulfate Hb, a cyanide-free method, at wavelength 543 nm [34]. The reagents required for the operation of the blood cell counter 33 were supplied by the SFRI Corporation. Because fish have nucleated RBCs, their WBC count is potentially hampered on many types of automated hematology analyzers [35]. A blood sample (20 μL) was added to 0.38 mL WBC diluting fluid in a clean test tube to achieve a final dilution of 1:19. A drop of the diluted blood was charged onto a Neubauer chamber and allowed to settle for 2 min. A 40× objective lens of light microscopes was used for total leucocyte counting in the four corner squares. The number of cells counted for each blood sample was multiplied by 50 to obtain the total WBC count per microliter of blood [36].

The excised gill and kidney specimens were rinsed in phosphate-buffered saline (PBS; pH 7.8) to thoroughly remove blood attached to the specimens and they were weighed before homogenization. Tissues were minced and homogenized in PBS with a glass homogenizer on ice, thawed at 2°C–8°C or frozen at −20 °C, and centrifuged at 2000–3000 rpm for approximately 20 min. A sandwich enzyme-linked immunosorbent assay was performed to measure the NKA level according to the instructions of the Bioassay Technology Laboratory, Biotech Co., Ltd., Shangai, China. All microtiter plates provided in the kits were precoated with an antibody (Ab) specific to NKA [37, 38].

To measure the NKA level, a 50 μL standard sample, a blank, and a 40 μL sample were added to each well. Immediately, 10 μL biotinylated detection Ab (the detection Ab in the NKA kit is a fish monoclonal Ab), working solution and 50 μL horseradish peroxidase were added to each well but not the blank control, mixed well, covered with a plate sealer supplied by the kit manufacturer, and incubated for 60 min at 37 °C. The sealer was removed, and the plate was aspirated and washed 5 times with wash buffer. The plate was blotted onto paper towels. Then, 50 μL substrate solution A and 50 μL substrate solution B were added to each well, and the plates were resealed and incubated for approximately 10 min at 37 °C in the dark. To terminate the enzyme reaction, 50 μL stop solution was added to each well. The blue color changed yellow immediately, and the optical density was determined using an automatic microplate reader (Bio-Rad, model iMark, Japan) at 450 nm within 10 min after adding the stop solution. The NKA levels in the gills and kidney were determined using the appropriate standard curves, and the data are expressed as ng mL^−1^ [37, 38].

### 2.5 Statistical analyses

All data are expressed as the mean ± standard deviation. Their normality and homogeneity were verified with Kolmogorov–Smirnov dan Levene's test before statistical analysis. All statistical analyses were performed using IBM SPSS version 21 (IBM Corp., Armonk, NY, USA). If the data did not meet the assumption of normality and homogeneity of variance, the data were log transformed. Statistical analysis of the data was performed using two-way ANOVA followed by Tukey's HSD post hoc comparison test to evaluate the effect of Hg at different salinities on SO, serum ion levels, hematological parameters, and NKA level. Differences were considered to be significant when the *p*-value was <0.05.

## 3 Results

**Figure 1:**
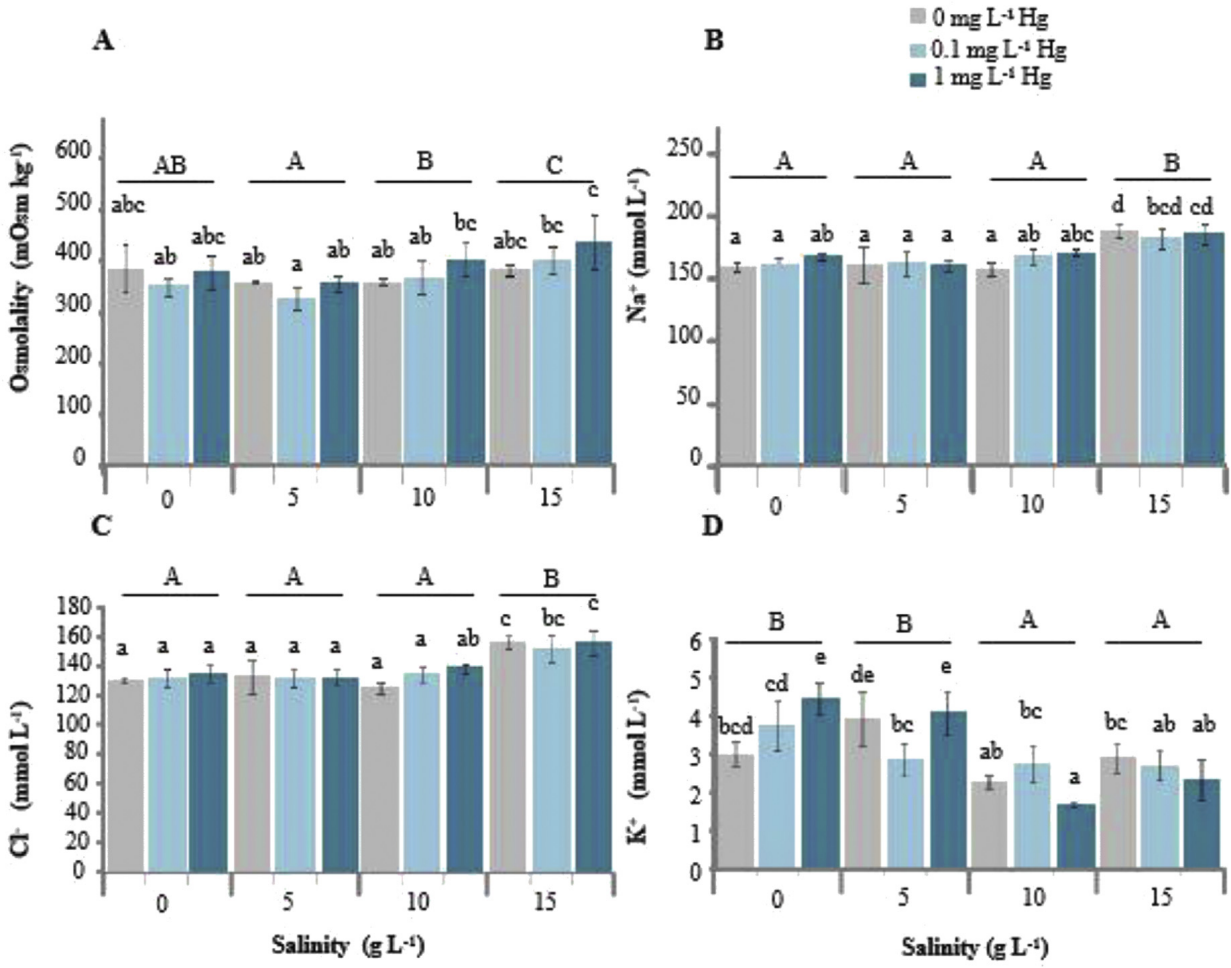
**A** Serum osmolality in *Oreochromis niloticus* exposed to mercury (Hg) and sacrificed after 96 h exposure. **B** Serum sodium **(**Na^+^) level. **C** Serum chloride (Cl^−^) level. **D** Potassium **(**K^+^) level. Lowercase letters indicate significant differences of values at different Hg concentrations, whereas uppercase letters indicate significant differences of values at different salinities. Mean values not sharing the same letter are significantly different (two-way ANOVA, Tukey's HSD post hoc comparison test, *p* < 0.05).

**Figure 2:**
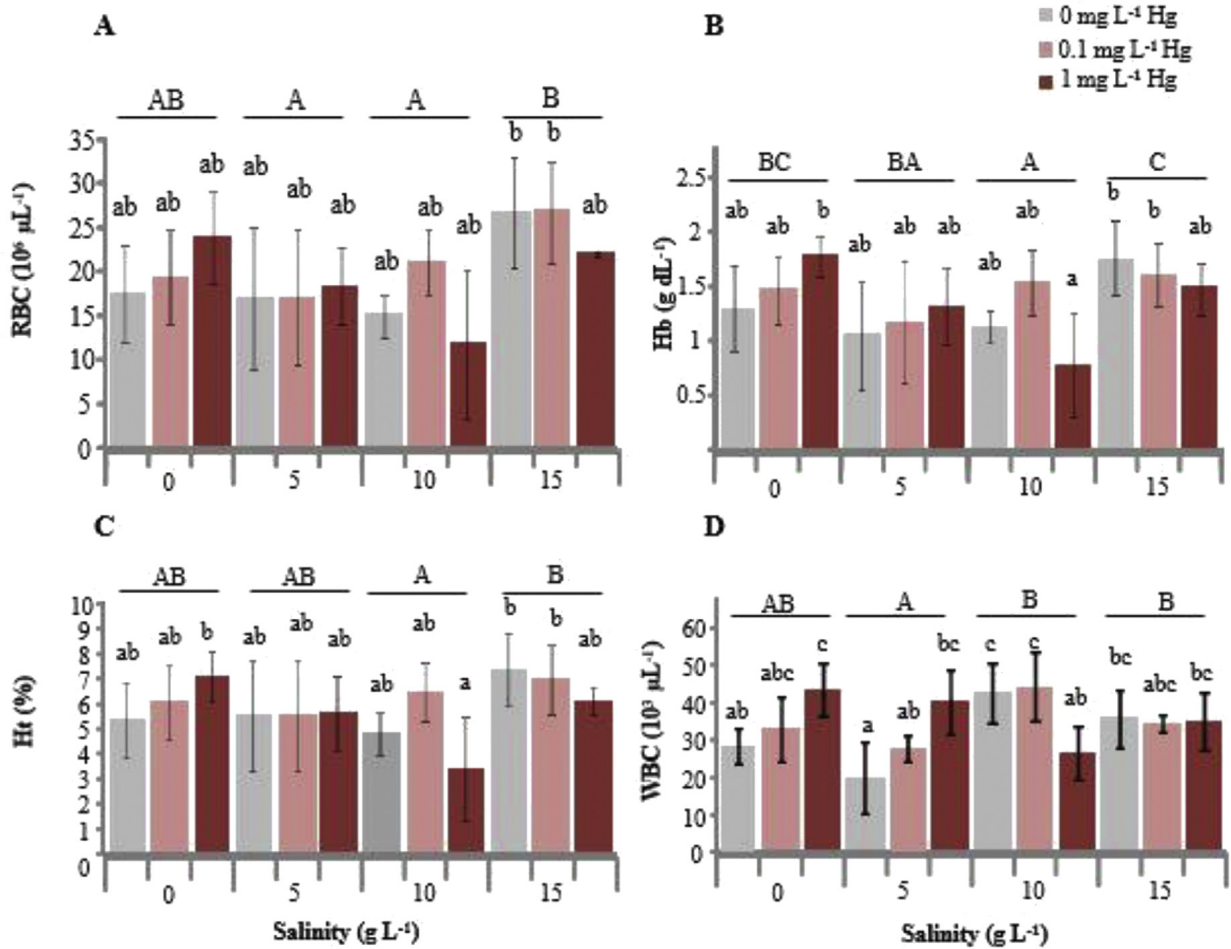
**A** Red blood cell (RBC) count in *Oreochromis niloticus* exposed to Hg and sacrificed after 96 h exposure. **B** Hemoglobin (Hb) level. **C** Hematocrit (Ht) level. **D** White blood cell (WBC) count. Lowercase letters indicate significant differences of values at different Hg concentrations, whereas uppercase letters indicate significant differences of values at different salinities. Mean values not sharing the same letter are significantly different (two-way ANOVA, Tukey's HSD post hoc comparison test, *p* < 0.05).

**Figure 3:**
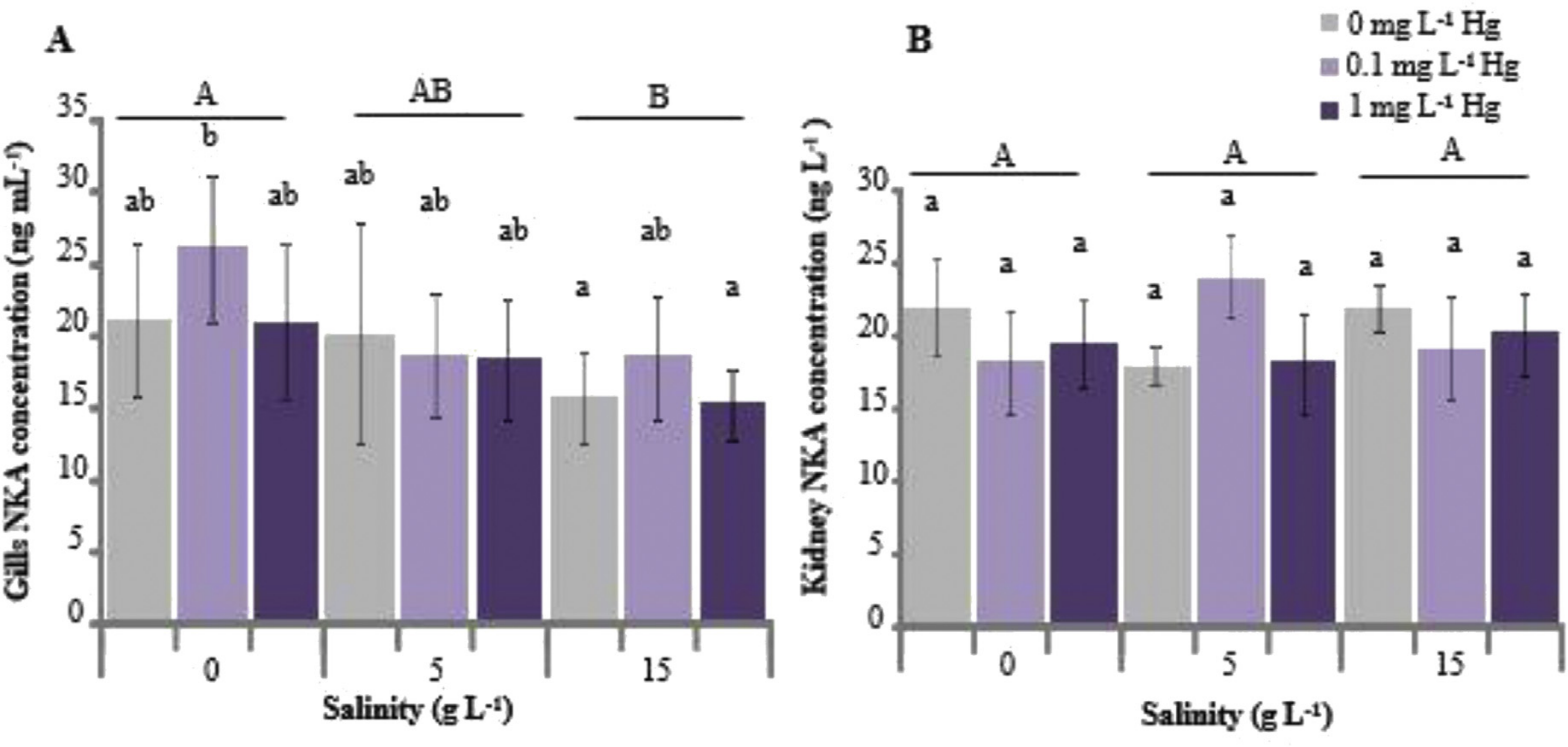
**A** Na^+^/K^+^-ATPase (NKA) level in the gills of *Oreochromis niloticus* exposed to Hg and sacrificed after 96 h exposure. **B** NKA level in the kidney of *O. niloticus* exposed to Hg and sacrificed after 96 h exposure. Lowercase letters indicate significant differences of values at different Hg concentrations, whereas uppercase letters indicate significant differences of values at different salinities. Mean values not sharing the same letter are significantly different (two-way ANOVA, Tukey's HSD post hoc comparison test, *p* < 0.05).

The effects of Hg and salinity on SO, Na^+^ level, chloride (Cl^−^) level, K^+^ level, Ht level, RBC count, Hb level, WBC count, and NKA level are presented in Figures Figure 1, Figure 2A–D and 3A, B, respectively.

### 3.1 SO

Two-way ANOVA revealed that there were significant effects of Hg (*p* = 0.003) and salinity (*p* = 0.000) on SO in tilapia; meanwhile, there was no significant Hg–salinity interaction (*p* = 0.151) in terms of SO (Table 1). SO was significantly higher at 10 and 15 g L^−1^ salinities than at 5 g L^−1^ salinity, but SO was significantly different at 10 g L^−1^ salinity compared with that of the 0 g L^−1^ salinity group. Regarding the different Hg treatments at the same salinity, SO in the control group was not significantly different compared with the 0.1 and 1 mg L^−1^ Hg exposure groups (Figure 1A). The highest SO was observed in the 1 mg L^−1^ Hg exposure group at 15 g L^−1^ salinity and the lowest in the 0.1 mg L^−1^ Hg exposure group at 5 g L^−1^ salinity.

**Table 1:** Two-way ANOVA for serum osmolality, serum ion levels, and hematological parameters after 96 h of exposure to Hg, salinity, and Hg–salinity interaction treatments.

Dependent variable	Source	F	*p*
Serum osmolality	Hg	6.634	0.003
	Salinity	10.405	0.000
	Hg–salinity	1.662	0.151
Serum sodium level	Hg	1.608	0.211
	Salinity	35.532	0.000
	Hg–salinity	1.614	0.164
Serum chloride level	Hg	2.085	0.135
	Salinity	38.001	0.000
	Hg–salinity	1.394	0.237
Serum potassium level	Hg	0.469	0.628
	Salinity	35.269	0.000
	Hg–salinity	9.549	0.000
Red blood cell count	Hg	0.735	0.485
	Salinity	6.190	0.001
	Hg–salinity	2.715	0.024
Hemoglobin level	Hg	0.985	0.381
	Salinity	4.162	0.011
	Hg–salinity	1.981	0.087
Hematocrit level	Hg	0.900	0.414
	Salinity	7.256	0.000
	Hg–salinity	1.792	0.121
White blood cell count	Hg	1.569	0.219
	Salinity	4.710	0.006
	Hg–salinity	9.326	0.000
Na^+^/K^+^-ATPase level (gills)	Hg	1.393	0.261
	Salinity	6.297	0.005
	Hg–salinity	0.603	0.663
Na^+^/K^+^-ATPase level (kidney)	Hg	0.898	0.416
	Salinity	0.135	0.874
	Hg–salinity	4.614	0.004

### 3.2 Serum ions

There were significant effects of salinity on serum Na^+^ (*p* = 0.000) and Cl^−^ (*p* = 0.000) levels in tilapia. However, the effects of Hg and Hg–salinity interactions on their levels were not significant (Table 1).

The Na^+^ levels in both the control and Hg exposure groups were significantly higher at 15 g L^−1^ salinity than at 0, 5, and 10 g L^−1^ salinities (Figure 1B). However, at the same salinity, the Na^+^ level did not significantly differ between the control and Hg exposure groups. The highest Na^+^ level was observed in the control Hg group at 15 g L^−1^ salinity.

The Cl^−^ level in both the control and Hg exposure groups was significantly higher at 15 g L^−1^ salinity than at 0, 5, and 10 g L^−1^ salinities; this was similar to the trend observed for the Na^+^ level (Figure 1C). Similarly, at the same salinity, the Cl^−^ level did not significantly differ between the control and Hg exposure groups. Similar to the Na^+^ level, the highest Cl^−^ level was observed in the control Hg group at 15 g L^−1^ salinity.

Significant effects of salinity (*p* = 0.000) and Hg–salinity interactions (*p* = 0.000) were found for serum K^+^ level in tilapia; however, the effect of Hg on the K^+^ level (*p* = 0.628) was not significant (Table 1). The K^+^ level in both the control and Hg exposure groups was significantly different at 10 and 15 g L^−1^ salinity compared with at 0 and 5 g L^−1^ salinities (Figure 1D). The K^+^ level between the control group and Hg exposure groups at the same salinity was significantly different at 0 and 5 g L^−1^ salinities. The serum K^+^ level in the 1 mg L^−1^ Hg exposure group was significantly different compared with the control group at 0 g L^−1^ salinity. The highest K^+^ level was observed in the 1 mg L^−1^ Hg exposure group at 0 g L^−1^ salinity, whereas the lowest K^+^ level was observed in the 1 mg L^−1^ Hg treatment at 10 g L^−1^ salinity.

### 3.3 Hematological parameters

According to two-way ANOVA, there were significant effects of salinity on RBC count, Hb level, and Ht level in tilapia. However, the effects of Hg and the Hg–salinity interactions on RBC count, Hb level, and Ht level were not significant (Table 1).

The RBC count in both the control and Hg exposure groups was significantly different at 15 g L^−1^ salinity compared with at 5 and 10 g L^−1^ salinities but was not significantly different at 0 g L^−1^ (Figure 2A). At the same salinity, the RBC count was not significantly different between the control and Hg exposure groups. The highest RBC count was observed in the 1 mg L^−1^ Hg exposure group at 0 g L^−1^ salinity, whereas the lowest RBC count was observed in the 0 mg L^−1^ Hg exposure group at 10 g L^−1^ salinity.

The Hb level in both the control and Hg exposure groups was not significantly different at 5, 10, and 15 g L^−1^ salinities compared with at 0 g L^−1^ salinity, but it was significantly different between 10 and 15 g L^−1^ salinities (Figure 2B). At the same salinity, the Hb level was not significantly different between the control and Hg exposure groups. The highest Hb level was observed in the 0 mg L^−1^ Hg exposure group at 15 g L^−1^ salinity, whereas the lowest Hb level was observed in the 0 mg L^−1^ Hg exposure group at 10 g L^−1^ salinity, similar to the trends observed for the RBC count and Ht level.

The Ht level in both the control and Hg exposure groups was significantly different at 15 g L^−1^ salinity compared with at 5 and 10 g L^−1^ salinities but was not significantly different at 0 mg L^−1^ salinity (Figure 2C). At the same salinity, the Ht level was not significantly different between the control and Hg exposure groups. The Ht level in all Hg exposure groups was not significantly different compared with the control group at all salinities. The highest Ht level was observed in the 0.1 mg L^−1^ Hg exposure group at 15 g L^−1^ salinity, whereas the lowest Ht level was observed in the 1 mg L^−1^ Hg exposure group at 10 g L^−1^ salinity, similar to that observed for the K^+^ level.

There were significant effects of the salinity (*p* = 0.006) and the Hg–salinity interactions (*p* = 0.000) on the WBC count. However, the effects of Hg on the WBC count (*p* = 0.219) were not significant. The WBC count in both the control and Hg exposure groups was not significantly different at 5, 10, and 15 g L^−1^ salinities compared with at 0 mg L^−1^, but it was significantly different at 5 g L^−1^ salinity compared with at 10 and 15 g L^−1^ salinities (Figure 2D). The WBC count in the control group compared with the Hg exposure groups at the same salinity was significantly different at 5 and 10 g L^−1^ salinities. The highest WBC count was observed in the 0 mg L^−1^ Hg exposure group at 10 g L^−1^ salinity, whereas the lowest WBC count was observed in the 0 mg L^−1^ Hg exposure group at 5 g L^−1^ salinity.

### 3.4 NKA level

Significant effects of salinity were observed on the NKA level in the gills; however, there were no significant effects of Hg, salinity, or Hg–salinity interactions on the NKA level in the kidney (Table 1).

The NKA level in the gills in both the control and Hg exposure groups was significantly different at 15 g L^−1^ salinity compared with at 0 g L^−1^ salinity but was not significantly different at 5 g L^−1^ salinity (Figure 3A). At the same salinity, the NKA level was not significantly different between the control and Hg exposure groups. The highest NKA level was observed in the 0.1 mg L^−1^ Hg exposure group at 0 g L^−1^ salinity, whereas the lowest NKA level was observed in the 0 mg L^−1^ Hg exposure group at 15 g L^−1^ salinity.

The NKA level in the kidney in both the control and Hg exposure groups was not significantly different at all salinities (Figure 3B). At the same salinity, the NKA level was not significantly different between the control and Hg exposure groups.

## 4 Discussion

Teleosts differ in their sensitivity and the nature of their responses to the effects of toxic metals [24]. In the present study, the effect of salinity on SO in tilapia was shown. Hg exposure causes differences in serum and plasma osmolality across different fish [39]. In most teleosts, Na^+^ and Cl^−^ account for at least 90% of blood osmolality, with the remaining being made up of ions such as K^+^ and Ca^2+^, proteins, and small organic molecules [40]. Osmoregulatory dysfunction is also related to Ht levels [41]; therefore, we examined the Ht level in this study.

Na^+^ and Cl^−^ levels significantly increased at the highest salinity, which indicates that in short-term exposure to Hg and salinity, only salinity had an effect on the Na^+^ and Cl^−^ levels. Because Na^+^ and Cl^−^ are the major ions in body fluid, their regulation is critical for osmoregulation [42]. In this study, fish could adapt to salinity and Hg changes, and therefore, their Na^+^ and Cl^−^ levels could gradually reach normal levels. In general, organisms at the larval stage are most sensitive to Hg exposure; methylmercury in fish is produced through bacterial methylation of inorganic Hg either in the environment or in bacteria associated with fish gills [43]. For more information related to fish gills, the NKA level was also measured. For example, Vernberg and O'Hara found that the highest Hg level in fish was in their gills [44].

The reduction in K^+^ level following exposure to 10 and 15 g L^−1^ salinities might occur to balance the osmotic difference in intracellular fluid caused by the increase in Na^+^, Cl^−^, and Ca^2+^ levels [45, 46]. Sanders and Kirschner [47] suggested that in a hyperosmotic environment, fish gills are permeable to K^+^ and that efflux is greater than influx. This means that a reduced K^+^ uptake, rather than an increased K^+^ loss, is a critical factor [48]. Thus, a decreased serum K^+^ level could be ascribed to osmotic adaptation [46].

At the same salinity, the RBC count was not significantly different between the control and Hg exposure groups. Simonato et al. [49] stated that within a 96 h exposure of fish to gasoline, a decrease in erythrocyte count could be observed. In contrast to previous studies, in this study, the erythrocyte count slightly increased at 10 and 15 g L^−1^ salinities in the Hg exposure groups compared with the control group, but not significantly. However, the RBC count decreased at 0 and 5 g L^−1^ salinities in the Hg exposure groups compared with the control group, but not significantly. This finding is in accordance with that reported by Shen et al. [50], who reported that erythrocytes are also associated with immune responses. In circulation, erythrocytes have a major role, i.e., in gas transportation to cells and tissues. In addition, erythrocytes support the immune response in fish.

The Hb level was not significantly different between the control and Hg exposure groups at 5, 10, and 15 g L^−1^ salinities compared with at 0 g L^−1^ salinity, but it was significantly different at 10 g L^−1^ salinity compared with at 15 g L^−1^. At the same salinity, the Hb level was not significantly different between the control and Hg exposure groups. The highest Hb level was in the 0 mg L^−1^ Hg exposure group at 15 g L^−1^ salinity, whereas the lowest Hb level was in the 0 mg L^−1^ Hg exposure group at 10 g L^−1^ salinity; this was similar to the trend observed for the RBC count and the Ht level.

In addition, the Hb level decreased due to the disruption of Hb biosynthesis. Hussan et al. [51] obtained a significant decrease in the oxygen consumption rate of Asian carp *Catla catla* with increasing Hg concentrations. A recent study stated that erythrocytes have a role in the immune system; both WBC and RBC counts in tilapia (*Oreochromis niloticus*) express similar immune response genes [50]. WBC count in both the control and Hg exposure groups was not significantly different at 5, 10, and 15 g L^−1^ salinities compared with at 0 g L^−1^ salinity, but it was significantly different at 5 g L^−1^ salinity compared with at 10 and 15 mg L^−1^ salinities. The highest WBC count was observed in the 0 mg L^−1^ Hg exposure group at 10 g L^−1^ salinity, whereas the lowest WBC count was observed in the 0 mg L^−1^ Hg exposure group at 5 g L^−1^ salinity.

According to Bond [52], the Ht level in teleosts ranges from 20% to 30% and is approximately 42% in some marine fish species. In this study, information regarding the effect of Hg exposure on Ht level is not shown because there was no difference after 96 h of exposure both at low and high Hg concentrations. In the present study, it was found that only salinity affected the Ht level in *O. niloticus*. Because the Ht level is related to osmoregulatory functions [41], SO, Na^+^, and Cl^−^ increase with increasing salinity of the media.

The increase in WBC count in this study is similar to that in the study by Soegianto et al. [21], which revealed that there was an increase in the number of tilapia leukocytes after exposure to salinity for 7 days. This increase can be attributed to nonspecific immune responses to stress to restore ionic balance as a result of prolactin–cortisol interactions [53].

At the same salinity, the NKA levels in both the gills and kidney were not significantly different between the control and Hg exposure groups. Meanwhile, the NKA level in the gills in both the control and Hg exposure groups was significantly different at 15 g L^−1^ salinity compared with at 0 g L^−1^ salinity. Dogan and Canli [54] observed that in response to chronic Hg exposure of *O. niloticus*, the activities of the gills’ NKA at 10 g L^−1^ increased. These characteristics and its general availability and economic laboratory maintenance make this fish an excellent model for future research into osmoregulation. In response to salinity challenges, the opposing patterns of the NKA level in the gills and kidney of tilapia could be attributed to the different roles of these two osmoregulatory organs [55].

## 5 Conclusion

Based on these results, it can be concluded that higher salinity reduced the effect of Hg on K^+^ level and WBC count in tilapia. Therefore, fish farmers should consider increasing the salinity up to 15 g L^−1^ during tilapia farming to avoid the long-term harmful effects of Hg.

## Declarations

### Author contribution statement

Kiki Syaputri Handayani: Conceived and designed the experiments; Performed the experiments; Analyzed and interpreted the data; Wrote the paper.

Bambang Irawan: Conceived and designed the experiments; Analyzed and interpreted the data.

Agoes Soegianto: Conceived and designed the experiments; Performed the experiments; Contributed reagents, materials, analysis tools or data; Wrote the paper.

### Funding statement

The Directorate General of Resources for Science, Technology and Higher Education of the Ministry of Research, Technology and Higher Education Republic Indonesia supported this work under the Sandwich-Like Program at University of Montpellier (under supervision of Prof. J.H. Lignot). Part of this study was also supported by a grant from 10.13039/501100008463Universitas Airlangga.

### Competing interest statement

The authors declare no conflict of interest.

### Additional information

No additional information is available for this paper.
